# Information‐seeking behaviour of primary care clinicians in Singapore at the point‐of‐care: A qualitative study

**DOI:** 10.1111/hir.12535

**Published:** 2024-05-28

**Authors:** Mauricette Moling Lee, Wern Ee Tang, Helen Elizabeth Smith, Lorainne Tudor Car

**Affiliations:** ^1^ Lee Kong Chian School of Medicine Nanyang Technological University Singapore Singapore; ^2^ Health and Social Sciences Singapore Institute of Technology Singapore Singapore; ^3^ Clinical Research Unit National Healthcare Group Polyclinics (NHGP) Singapore Singapore; ^4^ Department of Primary Care and Public Health, School of Public Health Imperial College London London UK

**Keywords:** Asia, south east, clinical guidelines, clinical questions, digital information resources, evidence‐based medicine, evidence‐based nursing, information literacy, internet access, Mobile health (M‐Health), primary care

## Abstract

**Background:**

Clinicians' information‐seeking behaviours impact patient care quality. Earlier studies indicated that barriers to accessing information deter clinicians from seeking answers to clinical questions.

**Objectives:**

To explore primary care clinicians' information‐seeking behaviour at point‐of‐care, focusing on when and how they seek answers to clinical questions.

**Methods:**

Semi‐structured interviews were conducted with 45 clinicians after clinical sessions to investigate their information‐seeking habits. Follow‐up interviews were conducted after a week for those intending to address unanswered queries.

**Results:**

Two thirds of clinicians encountered questions during care, with nearly three quarters resolving them during the session. Colleagues, guidelines and online platforms were common information sources, with smartphones being used to access Google, WhatsApp or UpToDate®. Facilitators included reliable sources and the drive to confirm knowledge, while barriers included ineffective search methods and high workload. Despite challenges, most clinicians expressed satisfaction with their information‐seeking process.

**Discussion:**

The findings underscore the increasing use of smartphones for accessing clinical information among Singaporean primary care clinicians and suggest the need for tailored training programmes and guidelines to optimise information‐seeking practices.

**Conclusion:**

Insights from this study can inform the development of training programmes and guidelines aimed at improving information‐seeking practices among primary care clinicians, potentially enhancing patient care quality.

**Key Messages**

- Regulations on searching the internet in Singapore public healthcare institutions may have affected information‐seeking behaviour among primary care clinicians. Almost two thirds of clinicians had questions about their clinical care, and most of them answered those questions in their clinical session.
- Clinicians are increasingly accessing clinical information on their smartphones using Google search, WhatsApp messaging or UpToDate®.
- Key facilitators for accessing clinical information include speed and the availability of reliable sources. The barriers include ineffective means of search, and unstable internet connections.
- Future training on information literacy for health professionals should consider the likely use of personal smartphones for access to evidence.

## INTRODUCTION

The search for, and use of information to meet information needs is known as information‐seeking behaviour (Braun et al., 2006; Clarke et al., 2013; Ely et al., 1992). Evidence shows that clinicians frequently engage in information‐seeking to meet their information needs by raising questions about patient care during their clinical practice. Yet, only around half of the clinicians pursue answers to their clinical questions (Del Fiol et al., 2014). The lack of time was reported as the main barrier to pursuing clinical answers (Brassil et al., 2017). In addition, the lack of formal training in the use of literature databases, little awareness of relevant information digital sources, and the lack of access to information at the point‐of‐care contribute to specialists and primary care clinicians not pursuing clinical answers (Brassil et al., 2017). As medical knowledge expands, along with the growing complexity of healthcare, it is important to improve information‐seeking behaviour of clinicians (Del Fiol et al., 2014).

Access to and use of electronic clinical sources has been shown to improve clinicians' knowledge and skills for their practice (Maggio et al., 2019). Healthcare professionals have access to a variety of electronic clinical information and evidence sources, including clinical practice guidelines (CPGs), clinical pathways, care pathways, websites, databases for medical literature, medical mobile apps and their colleagues. Healthcare providers use evidence differently, which may result in poorer health outcomes, such as poorer quality of care and patient satisfaction (Bruin‐Huisman et al., 2017; Cahir et al., 2014; Davies, 2007; Gill et al., 1996; Salisbury et al., 1998; Young & Ward, 2001). An approach commonly used to increase the adoption of evidence is the development of systematically developed evidence‐based recommendations such as CPGs, or care pathways. By using these structured, reliable, and consistent evidence‐based sources, unnecessary variation in clinical practice can be reduced (Scott et al., 2011). These sources, however, are expensive to develop and update, contextualise, and not universally adopted across health systems. In addition, CPG adoption is reduced by unsuitable presentation formats, clinicians' lack of time, CPG's perceived lack of reputation and relevance (Langley et al., 1998; Le et al., 2016). Clinicians at times turn to other clinical information sources which may be less reliable, trustworthy, or current compared to CPGs. Healthcare professionals working in Singapore's public healthcare institutions also faced the challenge of internet surfing separation because of breach of patient data privacy (Today, 2018). Internet surfing separation occurred when regulations on internet searches were imposed in Singapore public healthcare institutions (Ministry of Health, 2019). All clinic workstations are unable to connect to the internet. Only a few organisation workstations were given internet access, contingent on availability. The internet surfing separation does not affect clinicians' personal laptops or smartphones. In Singapore, in 2019 when this study was conducted, there was a reduction in internet access in the institutions which may have affected information‐seeking behaviour of clinicians; with work computers no longer providing access to online information resources. We decided to investigate if primary care clinicians were turning to other channels such as their smartphones to access clinical information and the best available recent research.

The lack of access to relevant information or the use of unreliable information can reduce the quality and safety of patient care provided in different healthcare settings, including primary care (Ely et al., 2002; Norlin et al., 2007). It is therefore important to explore information needs and information‐seeking behaviour in specific clinical settings. In this study, we aimed to explore and report on the information‐seeking behaviour of primary care clinicians working in polyclinics in Singapore. More specifically, we were interested in exploring:

1. Whether primary care clinicians had clinical questions arising from their clinical practice and whether they obtained answers to unanswered questions in or after consultation;
2. The type of information needs that the primary care clinicians required;
3. The type of sources clinicians used to meet their information needs;
4. The barriers and facilitators behind primary care clinicians' information‐seeking behaviour.

## METHODS

**FIGURE 1:**
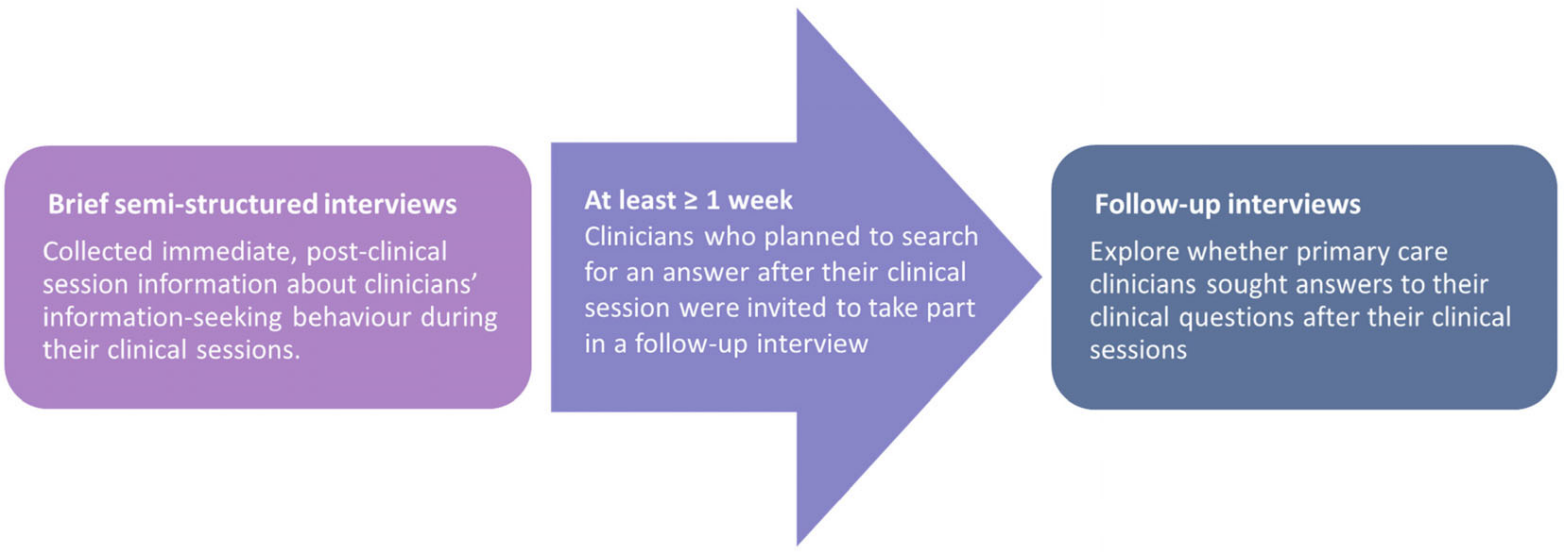
Flow diagram on the conduct of the interviews. [Colour figure can be viewed at wileyonlinelibrary.com]

This qualitative study was conducted between August 2019 and December 2019. We chose brief semi‐structured interview because issues of privacy prevented us from recording the clinical session. In clinical practice, this method is the least intrusive yet a consistent approach to real‐time data collection (Del Fiol et al., 2014; Gask et al., 2005). Brief semi‐structured interviews were conducted after the clinical sessions, and self‐reporting may be a good alternative to direct observations given the problematic logistics, patient confidentiality, and privacy issues (Del Fiol et al., 2014). Figure 1 presents the flow diagram on the conduct of the interviews. The follow‐up interview was conducted for eligible participants. No interviews were repeated.

This study was approved by the Review Board (Reference Number: 2018/01355). All participants read the study information sheet before providing written consent. This study followed the consolidated criteria for reporting qualitative research guidelines (Tong et al., 2007) (see Appendix 1).

### Participants and recruitment

We included a convenience sample of primary care doctors and registered nurses working in the polyclinics, aged ≥21 years and who were fluent in English. While convenience sampling is not the most rigorous method of sampling and reduces the generalisability of the findings, it can be a practical alternative when conducting exploratory studies when time, funding, or resources to conduct interviews are limited (Stratton, 2021). Primary care in Singapore is provided through a network of publicly funded polyclinics and private outpatient clinics across the island (Ministry of Health, 2022). There are 23 polyclinics and over 1800 general practitioner clinics (Ministry of Health, 2022). Private general practitioners provide primary care to around 80% of the population (Ministry of Health, 2022). Three healthcare organisations form the polyclinics: National Healthcare Group, National University Health System and SingHealth. These polyclinics served various communities in the central, northern, north‐eastern, western and eastern areas of Singapore. In this study, primary care clinicians were recruited from publicly funded polyclinics serving much of the central and northern Singapore's population while having reduced internet access, the generalisation of the results can be limited. The information‐seeking behaviour of clinicians working in those polyclinics may be affected. The clinicians were recruited via personal contacts and advertisements. Participants had to be available for interview after a clinical session. Five potential participants were contacted but did not reply to the invitation; two potential participants declined to participate in this study but did not explain why. One potential participant resigned before the study began and thus did not participate.

For the second part of the study, participants with unanswered clinical questions and who had consented to follow up, were contacted to explore their subsequent information‐seeking behaviour.

There were no patients engaged in the conception or execution of this study.

### Data collection

All participants of the brief semi‐structured interview were available to participate in face‐to‐face interviews. The interviews were conducted by a female researcher (MLM) in designated private meeting rooms or consultation rooms at the involved research sites. Before the start of the interview, MLM introduced herself, stated the aim of the interview, explained confidentiality, obtained informed consent and permission to use a digital voice recorder.

MLM conducted the interview with an interview guide (see Appendix 2). The interview guide was developed after reviewing the relevant literature and research team discussions (Aakre et al., 2019). It included questions relating to the participants' information‐seeking during the recently completed clinical session. Questions included the type and number of questions asked, pursued and answered, time spent searching for answers, the information sources used and the perceived facilitators and barriers to seeking information. The interview sessions were digitally recorded and transcribed. Field notes were taken during the interviews. Data saturation, defined as no new themes arising after three consecutive interviews (Saunders et al., 2018), was achieved after 45 interviews, we did not recruit further. Of the 21 nurses, one did not conduct any clinical sessions and so we were unable to proceed the interview with that nurse. Participants were compensated with a SGD15 voucher and a meal upon completion of the interview.

All follow‐up interviews were conducted by MLM over the telephone in a private meeting room at Lee Kong Chian School of Medicine, Nanyang Technological University, Singapore or in designated private meeting rooms in the clinical setting. Before the start of the interview, MLM introduced herself, stated the aim of the interview, explained confidentiality, obtained permission to use a digital voice recorder. MLM then proceeded with the follow‐up interview with an interview guide (see Appendix 3). The interview guide consists of questions relating to the participants' information‐seeking unanswered clinical questions after their clinical session. Questions covered topics such as the time spent searching for answers, the information sources used and the perceived barriers and facilitators to searching for information. Field notes were taken during the interviews.

### Data analysis

The qualitative data were analysed using Burnard's method, a structured approach to thematic content analysis established in 1991 (Burnard, 1991). To discern the various types of questions, we conducted an analysis using a well‐regarded classification framework for clinicians' clinical inquiries, as proposed by Ely et al. (1992). First, two researchers (MLM and LTC) familiarised themselves with the transcripts by reading them multiple times. Second, the initial codes with definitions were proposed. Third, the themes were derived from the codes. Fourth, the researchers discussed and combined their themes for comparison. Finally, they reached a consensus on the themes to be used and how to define them (see Appendix 4). A third reviewer, HES acted as an arbiter. The coding of transcripts was performed using a word processor.

### Researcher reflexivity

In this qualitative research, reflexivity is recognised (Olmos‐Vega et al., 2023)—critical analysis of our roles, potential bias and influence on the research process, including formulation of research questions, data collection and analysis. This study aimed to enhance transparency and trustworthiness by weaving reflexive reporting practices throughout the manuscript. Examining the most important decisions and dynamics in the research process, emphasising personal, interpersonal, methodological, and contextual aspects (Olmos‐Vega et al., 2023). For example, in terms of methodological reflexivity, we detailed how we determined that the data was saturated. In addition, to substantiate the personal and methodological components of reflexivity, we provided insights into features of the study population and setting that are especially relevant to help readers judge the transferability of the study's findings to other circumstances in the subject to discussion. The use of reflexive journals in this study documents personal reflections and biases including the navigation of biases. Excerpts from the journals can be found in Appendix 6.

## RESULTS

### Overview

A total of 45 clinicians were recruited, 25 primary care doctors and 20 nurses. All nurses and eight doctors who participated in this study were females. Doctors had a range of one to five clinical questions with a median of two clinical questions in their preceding clinical sessions. While nurses had a range of 1–10 clinical questions with a median of one clinical question. Each interview session lasted not more than 20 min.

**FIGURE 2:**
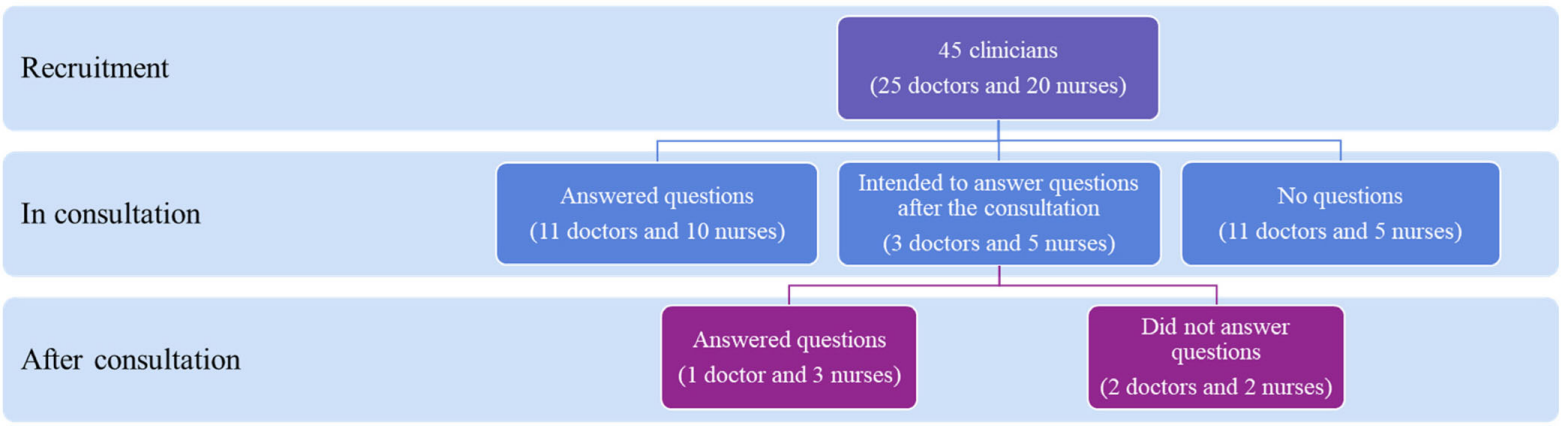
Number of answered and unanswered clinical questions by the clinicians. [Colour figure can be viewed at wileyonlinelibrary.com]

As shown in Figure 2, 29 (64%) clinicians had clinical questions in their consultation. Of which, 11 (44%) doctors and 10 (50%) nurses answered their questions within the consultation, and three (12%) doctors and five (25%) nurses had unanswered questions. Another 11 (44%) doctors and 5 (25%) nurses did not have clinical questions within the consultation.

Three doctors and five nurses who had unanswered questions within the consultation, had the intention to seek answers to their clinical questions. We found that one (33%) doctor and three (60%) nurses answered their questions after consultation and the remaining two (67%) doctors, and two (40%) nurses did not. Since clinicians considered patients to be their source of information, many of their questions went unanswered as patients missed their scheduled appointments or refused to follow treatment plans. Only one (4%) of the 25 doctors and four (20%) of the 20 nurses who began looking for clinical answers went on to investigate other disease types or specialty areas. The level of satisfaction among 12 doctors and 13 nurses who sought answers to their clinical questions are presented in Appendix 4. Most clinicians felt satisfied looking for answers to their questions, with the information they had found, and their information‐seeking process. None of the clinicians were very dissatisfied.

### Thematic analysis

**FIGURE 3:**
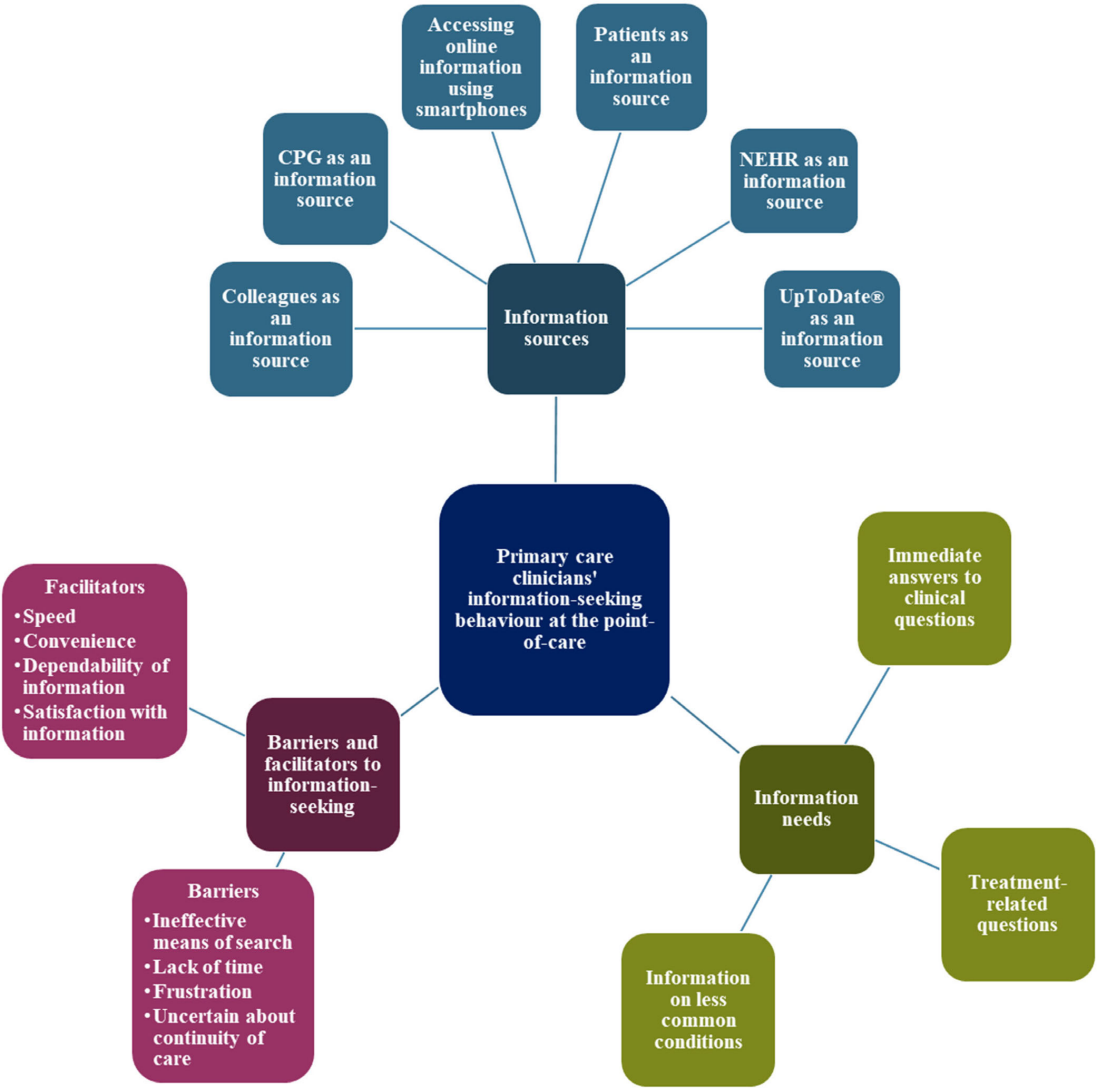
Themes and subthemes arising from the interviews. [Colour figure can be viewed at wileyonlinelibrary.com]

The themes were grouped into broader categories namely, (1) information sources, (2) information needs and (3) barriers and facilitators to information‐seeking. This is represented in Figure 3 (see Appendix 5).

#### Information sources

##### Colleagues as an information source

There were several sources of information that clinicians used at the point‐of‐care. It was reported that clinicians relied primarily on colleagues for information, but factors such as proximity, availability, the years of experience the colleague had, and whether that colleague was a specialist affected clinicians' decisions to approach them for information.

‘…we do call our own colleagues…we do have doctors [who] are quite senior. With more than 30 years of experience in the clinic. So, we do call the seniors’. Doctor08.

‘I think the reason why going through colleague is my first way of seeking information is because it's the most convenient. It's the laziest method. I mean, they're just next door. You just ask them’. Nurse02.

##### CPGs as an information source

Another common source of information is the use of online sources. Some clinicians commonly refer to their organisation's online protocols and renowned medical websites for clinical information. CPGs were reported as the second most frequently used clinical source by nurses for answering clinical questions.

‘I actually referred to the Ministry Guidelines because we have the hardcopy at our desk’. Nurse21.

‘Maybe if local context. Because it can be quite limited, [with] the kind of information you can find on the in‐house medical centre website. Then maybe I will try and see online. Those US medical centres tend to have more information on their website’. Nurse02.

‘I get the answer from the email, because the latest guideline was from the email’. Nurse15.

##### Accessing online information using smartphones

Clinicians commonly use their smartphones to access clinical information. For example, using the Google search engine for quick answer to their clinical question.

‘…when you Google, is there a specific website or link? no. I usually just look, browse through the first three or four hits’. Doctor26.

‘Sometimes when I on the train…I will take out my phone, then I will Google’. Nurse16.

##### Patients as an information source

Clinicians also saw patients as a source of information. For instance, one nurse mentioned reading patients' online reviews on their clinical management, and doctors awaited patients to return for their next clinical appointment to discuss patients' treatment plan.

‘I have to decide or make a diagnosis or come to a treatment plan based on the information they give’. Doctor05.

‘…if from the internet only, maybe the reviews, whether…It has helped them’. Nurse01.

##### National Electronic Health Record as an information source

Clinicians found accessing National Electronic Health Record (NEHR) for clinical information more efficient than having discussions with colleagues provided the internet connection was stable.

‘NEHR is much faster compared to calling someone to get the information’. Doctor06.

‘I can get it from my national registry where I can see all the records depends on the internet connection if they are very fast then five minutes I can get it clear’. Nurse07.

##### UpToDate® as an information source

Doctors reported that the access to UpToDate® online source was provided by their organisation, easily accessible via organisation's network, and was also their second most used source for answering clinical questions.

‘…why did you choose UpToDate®? easy access. We can access through intranet’. Doctor30.

Doctors also mentioned that UpToDate® provided more insights on drugs than the organisation's drug compendium.

‘…my initial search was…Within the drug compendium then I had to move on to UpToDate®…So that I could have a more detailed information because the drug compendium did not give me much clarity. Or did not allow me to make a decision straight‐forward’. Doctor05.

Doctors were aware that UpToDate® was also available in mobile app version.

#### Information needs

##### Immediate answers to clinical questions

Doctors frequently reported that they needed immediate answers to diagnosis and treatment‐related questions, while nurses frequently sought answers to management and treatment‐related questions.

‘Whether symptoms were significant, whether any clinical signs are missed out, whether any significant history to note’. Doctor01.

‘…whether the referral was made to see a specialist after I refer to the doctor for any abnormal findings that I see today’. Nurse13.

##### Treatment‐related questions

The most common type of questions among clinicians were treatment‐related questions. These questions were aimed at gathering in‐depth understanding of the patients' conditions.

‘Whether this drug is. Indicated for certain use, safe for pregnancy, common dosages’. Doctor24.

‘I think these (*clinical questions*) are more of in depth knowledge base. Not the scraping the surface, then we tell the patient’. Nurse16.

##### Information on less common conditions

Doctors tended to have clinical questions when managing patients with special considerations, such as, needing to switch the medications.

‘…today morning I had to see a pregnant lady who was on anti‐hypertensives. I had to switch anti‐hypertensives, which is not very oftenly done within the polyclinic clinic setting’. Doctor05.

#### Barriers and facilitators to information‐seeking

##### Barriers to information access

The barriers to accessing information included ineffective means on search. For instance, clinicians reported that the poor user interface of the medical websites affected the speed of information retrieval. Also, the unstable internet access limited their access to clinical information.

‘…whatever I can find from UpToDate® about the latest management. Actually, even that took a while because the website was not very. It's not that compact. You have to go and read through a long list of things. So, about ten minutes’. Doctor09.

‘…if it's fast that means internet connection very fast, it's all good. If not, it's frustration. Sometimes disappointed if you try to get it and it just won't work system down’. Nurse07.

Doctors mentioned that they did not have the capacity to consider clinical questions, due to the multiple duties they had at work.

‘Today I was too busy, so, I didn't had time to think that much’ Doctor31.

‘…juggle the timing because they've already put the patients on my list the patient already outside my room. But I still have other duties to do I can't really do both the same time’. Doctor11.

Some clinicians reported feeling pressured and frustrated at work when they were unable to answer their clinical questions. However, these feelings also spurred clinicians to seek answers.

‘…there's a bit of frustration. Because patients sometimes expect you to know everything’. Doctor06.

‘…it can start if it's fast that means…internet connection very fast, it's all good. If not, it's frustration. Sometimes disappointed if you try to get it and it just won't work system down’. Nurse07.

Some clinicians struggled to understand our questions about their information‐seeking behaviour (see Appendix 2) and asked for further clarifications.

‘For the patient care? Like clarify doubts with colleagues and everything you mean specific questions which I didn't know the answer or I thought I know the answer’. Doctor01.

‘Any question as in is there any restriction? Any sort of question is it?’ Nurse02.

Nurses also mentioned the lack of continuity of care affecting nurses' information‐seeking as they were unsure of doctor's treatment plan for patients. Hence, uncertain about what information to seek about patients' condition.

‘And I feel like based on my own part what I can do is only to refer you to the doctor but honestly speaking, I'm not sure what is really being done, the continuity of care subsequently. Yeah. I'm not sure it's, like, a doubt that I have yet to really go and address’. Nurse02.

##### Facilitators to information access

The facilitators to accessing information included speed and convenience.

‘…today it was very fast less than five minutes. It was sort of urgent’. Doctor01.

In addition to the speed of access and convenience, doctors reported that the dependability of information was the next most important factors in their clinical source selection.

‘…we do call our own colleagues we have doctors are quite senior. With more than 30 years of experience in the clinic. So, we do call the seniors’. Doctor08.

While nurses reported that, being eager to confirm pre‐existing knowledge were equally important in obtaining clinical answers.

‘…the doctor is knowledgeable. But we still have to make sure that the things we've done are in line with the clinical’ Nurse05.

Most clinicians expressed feeling satisfied when retrieving answers to their clinical questions.

‘Satisfaction. I am happy that we got the result’. Doctor25.

‘…fast, is good, you got certainty immediately and then you've got satisfaction’. Nurse07.

## DISCUSSION

Our study explored information‐seeking behaviour, and immediate information needs at the point‐of‐care among primary care clinicians in Singapore. Two thirds of clinicians in our study had about one clinical question during their clinical session. Approximately half of them answered their questions during their clinical sessions. Clinicians considered patients to be their source of information, so many of their questions went unanswered because patients missed appointments or refused to follow treatment plans. Clinicians commonly sought treatment‐related questions. They commonly used Google search engines, CPGs, and online medical sources such as UpToDate®. The facilitators of accessing information included the speed, the availability of reliable sources, and the drive to confirm pre‐existing knowledge. The barriers of accessing information included ineffective means of search, unstable internet access, and high workload. Most clinicians were feeling satisfied looking for answers to their questions, with the information that they found, and with their information‐seeking process.

We observed that clinicians were at times either unable to recall their information‐seeking behaviour or were unfamiliar with the concept. Identifying information needs and translating this need into answerable clinical questions is the first essential step in information‐seeking (Geddes, 1999; He et al., 2018). This information‐seeking process is the most important and difficult step in evidence‐based medicine (EBM) (Kang, 2016). EBM promotes the use of current best evidence in making appropriate clinical decisions taking patients' values and preferences into consideration (Kang, 2016). We found that clinicians commonly specified patients as an important source of information. This is consistent with a study that identified patient information as the most important type of information (Ayatollahi et al., 2013) The source of information in EBM should be current high‐quality research that is applicable to specific patients (Rosenberg et al., 1998). Clinical questions developed for EBM should also reflect patients' unique circumstances and be answerable by clinicians. While collection of patient‐related information within a clinical session was critical in connecting current best evidence with patients' values and preferences, the result of unanswered clinical questions should not be solely dependent on patients as an information source. Some clinicians in our study struggled with these concepts suggesting towards a need for further training or a refresher in the use of EBM for clinical practice. Moreover, the fact that clinicians were mostly satisfied with their information‐seeking process may suggest that they are uninformed of their limitations in their information‐seeking skills. However, besides the necessity for EBM skills, the challenges associated with the feasible use of acquired information, such as the appropriateness of information to the different and unique conditions in family practice, persist (van der Keylen et al., 2020).

Clinicians in our study expressed frustration when they experience unstable connectivity in accessing clinical information on the organisation's internet network. Doctors also faced challenges when using organisation‐provided UpToDate® web‐based clinical resource reporting that it was too wordy and hard to navigate. Research shows that when doctors felt positive about seeking clinical information, they were motivated to seek answers to their clinical questions (Dawes & Sampson, 2003; Del Fiol et al., 2014; González‐González et al., 2007). In addition, clinicians commonly use their smartphones to access clinical information. This contributes to one of the six healthcare improvement goals: shortening the time between diagnosis and treatment (Institute of Medicine Committee on Quality of Health Care in America, 2001). To mitigate the feelings of frustration and improve the positiveness in information‐seeking among clinicians, the provision of evidence‐based point‐of‐care clinical sources, should be easily accessible, user‐friendly and reliable (Aakre et al., 2019; Daei et al., 2020; Maggio et al., 2019). It is imperative that focus is placed on enhancing the abilities and knowledge of clinicians by providing them with the necessary infrastructure, support and training (Neves & Burgers, 2022). As such, future interventions should train clinicians on accessing point‐of‐care resources, allow time for information‐seeking in practice, and improve user‐friendliness of existing resources. In addition, the development of certification standards for digital health resources that support person‐centred care, improve user experience and accounts for suppliers' responsibility for the standard of the resources may be useful in the clinical setting (Krist et al., 2021; Neves & Burgers, 2022).

Our study has both strengths and limitations. Our strengths include interviewing a large sample of participants, including both doctors and nurses (Ritchie et al., 2003) using a minimally intrusive and highly consistent approach to real‐time data collection in clinical practice (Del Fiol et al., 2014; Gask et al., 2005). The researchers were provided with sufficient details, sources, and exposure to courses on qualitative research and conducting telephone interviews before study commencement.

Some of our clinicians had difficulty recalling when they sought information during their clinical sessions. In future research direct observations may allow for collection of more accurate data less prone to recall bias (Del Fiol et al., 2014). We used a convenience sample of primary care clinicians from the public healthcare institutions, and we recognise that our findings may not be generalisable to those working in the private sector in Singapore. We wanted to protect participants' privacy and did not collect age information, but we recognised that this information would allow for more in‐depth research. The public and patients were not recruited for this study, which would have been valuable for exploring in more depth the diversity of healthcare concerns and potential ethical challenges primary care clinicians face daily (Neves & Burgers, 2022). For future work, we may consider employing co‐design to be sure that solutions take into account every aspect of the healthcare system, including patient experiences, clinical processes and technology feasibility (Glenn et al., 2015). Co‐design allows engagement between clinicians and patients in the design and implementation of health initiatives. Hence, recognise that patient viewpoints are critical for developing successful and user‐friendly solutions. Therefore, Co‐design may help to ensure that digital solutions in primary care meet the needs and preferences of patients as well as clinicians.

## CONCLUSION

In conclusion, almost two thirds of primary care clinicians in our study had questions about the clinical care they were providing, and more than half managed to answer their questions during the clinical session. Clinicians mentioned several important facilitators and barriers when accessing relevant information. Our findings can be used to inform the development of relevant training programmes and guidelines on information‐seeking practices. Future research should explore information needs and information‐seeking behaviour in a larger sample and include private primary care clinicians in Singapore and gather demographic data on participants, particularly their ages. Future research should also examine employing a co‐design approach with primary care clinicians and patients, in which patients can be involved in designing and maintaining digital solutions, particularly the usage of smartphones during consultations.

## FUNDING INFORMATION

This study is funded by National Healthcare Group‐Lee Kong Chian School of Medicine, Centre of Primary Health Care Research and Innovation Seedcorn Grant.

## CONFLICT OF INTEREST STATEMENT

The authors declared that they have no competing interests.

## ETHICS STATEMENT

This study has been reviewed for ethics approval.

## Supporting information


**Appendix S1.** Supporting Information.

**Appendix S2.** Supporting Information.

**Appendix S3.** Supporting Information.

**Appendix S4.** Supporting Information.

**Appendix S5.** Supporting Information.

**Appendix S6.** Supporting Information.
