# Supplementary material for: Information‐seeking behaviour of primary care clinicians in Singapore at the point‐of‐care: A qualitative study
Source: Health Info Libr J. 2024 May 28;41(4):418–28. doi: 10.1111/hir.12535 (PMC11649596; doi:10.1111/hir.12535)
Supplement: Supplementary file 2 — Appendix S2. Supporting Information. [file HIR-41-418-s006.docx]

| **Subject ID:** | SI | DR or NR |  |  |  |  |
| --- | --- | --- | --- | --- | --- | --- |


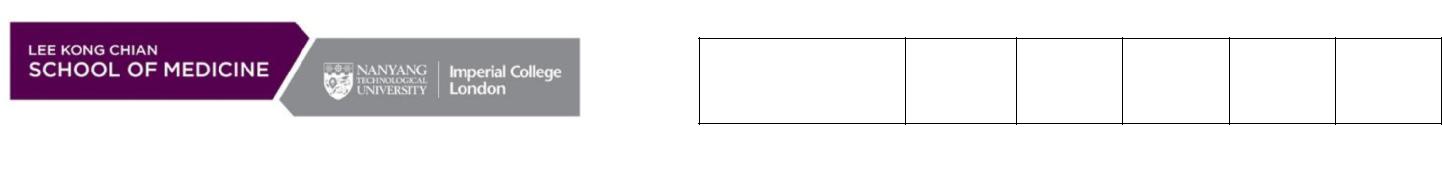


Structured interview Doctor or nurse Interviewer Initials Sequential number

**Protocol Title:**Information seeking behaviour of primary care practitioners in Singapore:

finding evidence to support high-quality patient care

**Structured Interview Guide**

**Introduction**

We are interested in exploring your health information seeking behaviour during clinical sessions. This 12-item interview will take no more than 20 minutes to answer. The interview will be recorded for all participants but pseudonyms are used for the purpose of transcription, analysis and publication. We may need to contact you again the future with a few more questions. All information obtained in this interview is strictly confidential and will be kept securely by the study team at the end of the study for 6 years and disposed of according to the Personal Data Protection Act. Please tick the appropriate box below to give your consent for us to use the information in this questionnaire for our study.

**Q1. How many questions about patient care did you have in this clinical session?**

|  |
| --- |

**Q2. What were your questions about patient care in this clinical session?**

|  |
| --- |

**Q3. Did you manage to pursue the answers to any of the questions about patient care you had in the clinical session just now?**

| ☐ Yes (please proceed to Q5) | ☐ No | ☐ Partially |
| --- | --- | --- |

**If your answer was no or partially, can you please explain why?**

|  |
| --- |

**Q4. If you did not search for an answer to your question about patient care, do you intend to search for it at some other time? When?**

|  |
| --- |

**Q5. If you answer yes to question Q3, how much time did you spend looking for an answer to your question(s) about patient care?**

| ☐ < 5 minutes |  |  |
| --- | --- | --- |
| ☐ 5 – 10 minutes |  |  |
| ☐ 11 - 20 minutes |  |  |
| ☐ > 20 minutes |  |  |

**Q6. How urgently do you require an answer to your question(s) about patient care?**

| ☐ Immediately |
| --- |
| ☐ Same day |
| ☐ Same week  ☐ It is not urgent |

**Q7. Where did you look for answers to your questions about patient care? Please tick all applicable options.**

☐ Discussion with colleagues

☐ Online search engines (e.g. Google)

☐ Online literature databases (e.g. PubMed)

☐ Evidence-based clinical decision support resource (e.g. Up-to-date)

☐ Clinical practice guidelines

☐ Institutional protocols or guides

☐ Textbooks

☐ Cochrane library

☐ Medical journals

☐ Drug compendium

☐ Other sources. Please specify: ______________________

**Q8. Why do you use the abovementioned information sources?
Please tick all applicable options.**

☐ Clarity of source

☐ Confirmation of pre-existing knowledge

☐ Convenience

☐ Speed of access

☐ Being able to share experiences

☐ To get support

☐ Reliability of information obtained (credible source)

☐ Other reasons. Please specify: _____________________________

**Q9. Could you please share if you started out searching for information in a particular area but moved on to explore other disease types/specialty areas:**(E.g. you started out searching for rosacea but ended up looking for information on eczema)

| **Initial search areas** | **Final search areas** |
| --- | --- |
|  |  |

**Q10. Please tick from the following list all applicable options describing the feelings you experienced while retrieving information:**

☐ Disappointment ☐ Frustration ☐ Confusion

☐ Trust ☐ Easiness ☐ Optimism

☐ Certainty ☐ Doubts ☐ Uncertainty

☐ Satisfaction ☐ Other feelings (please specify): _______________________

**Q11. How satisfied were you with the information you found?**

1 (Very dissatisfied) 2 (somewhat dissatisfied) 3 (Neither satisfied nor dissatisfied) 4 (Somewhat dissatisfied) 5 (Very satisfied)

**Q12. How satisfied were you with your information seeking process?**

1 (Very dissatisfied) 2 (somewhat dissatisfied) 3 (Neither satisfied nor dissatisfied) 4 (Somewhat dissatisfied) 5 (Very satisfied)

**Thank you for your time and effort in participating in this interview.**
