# Supplementary material for: Information‐seeking behaviour of primary care clinicians in Singapore at the point‐of‐care: A qualitative study
Source: Health Info Libr J. 2024 May 28;41(4):418–28. doi: 10.1111/hir.12535 (PMC11649596; doi:10.1111/hir.12535)
Supplement: Supplementary file 3 — Appendix S3. Supporting Information. [file HIR-41-418-s003.docx]

| **Subject ID:** | FUI | DR or NR |  |  |  |  |
| --- | --- | --- | --- | --- | --- | --- |


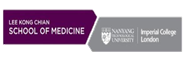


Follow-up interview Doctor or nurse Interviewer Initials Sequential number

**Protocol Title:**Information seeking behaviour of primary care practitioners in Singapore:

finding evidence to support high-quality patient care

**Structured Interview Guide – Follow Up**

**Introduction**

We are interested in exploring any change to your health information seeking behaviour during clinical sessions. This 8-item interview will take no more than 20 minutes to complete. All information obtained in this interview is strictly confidential and will be kept securely by the study team at the end of the study for 6 years and disposed of according to the Personal Data Protection Act.

**Q1. Since our meeting, did you manage to pursue the answers to any of the questions about patient care you had in the clinical session that morning?**

| ☐ Yes (please proceed to Q2) | ☐ No | ☐ Partially |
| --- | --- | --- |

**If your answer was no or partially, can you please explain why?**

|  |
| --- |

**Q2. If you answer yes to question Q1, how much time did you spend looking for an answer to your question(s) about patient care?**

| ☐ < 5 minutes |  |  |
| --- | --- | --- |
| ☐ 5 – 10 minutes |  |  |
| ☐ 11 - 20 minutes |  |  |
| ☐ > 20 minutes |  |  |

**Q3. Where did you look for answers to your questions about patient care? Please tick all applicable options.**

☐ Discussion with colleagues

☐ Online search engines (e.g. Google)

☐ Online literature databases (e.g. PubMed)

☐ Electronic evidence-based clinical decision support resource (e.g. Up-to-date)

☐ Clinical practice guidelines

☐ Institutional protocols or guides

☐ Medical journals

☐ Cochrane library

☐ Textbooks

☐ Drug compendium

☐ Other sources (please specify): ______________________

**Q 4. In reference to the above question, why do you normally use those particular information sources? Please tick all that apply.**

- Convenience
- Clarity of source
- Speed of access
- Reliability
- Familiarity with the source
- Comprehensiveness of available information
- Common use among colleagues
- Other reasons. Please specify: ___________________________

**Q5. Could you please share if you started out searching for information in a particular area but moved on to explore other disease types/specialty areas:**(E.g. you started out searching for rosacea but ended up looking for information on eczema)

| **Initial search areas** | **Final search areas** |
| --- | --- |
|  |  |

**Q6. Please tick from the following list all applicable options describing the feelings you experienced while retrieving information:**

☐ Disappointment ☐ Frustration ☐ Confusion

☐ Trust ☐ Easiness ☐ Optimism

☐ Certainty ☐ Doubts ☐ Uncertainty

☐ Satisfaction ☐ Other feelings. Please specify: _______________________

**Q7. How satisfied were you with the information you found?**
1 (Very dissatisfied) 2 (somewhat dissatisfied) 3 (Neither satisfied nor dissatisfied) 4 (Somewhat dissatisfied) 5 (Very satisfied)

**Q8. How satisfied were you with your information seeking process?**

1 (Very dissatisfied) 2 (somewhat dissatisfied) 3 (Neither satisfied nor dissatisfied) 4 (Somewhat dissatisfied) 5 (Very satisfied)

**Thank you for your time and effort in participating in this interview.**
