# Supplementary material for: Information‐seeking behaviour of primary care clinicians in Singapore at the point‐of‐care: A qualitative study
Source: Health Info Libr J. 2024 May 28;41(4):418–28. doi: 10.1111/hir.12535 (PMC11649596; doi:10.1111/hir.12535)
Supplement: Supplementary file 4 — Appendix S4. Supporting Information. [file HIR-41-418-s005.docx]

**Themes**

| **Primary care clinicians’ information-seeking behaviour at the point-of-care** | | |
| --- | --- | --- |
| **Information sources** | **Information needs** | **Barriers and facilitators to information-seeking** |
| Colleagues as an information source | Immediate answers to clinical questions | Facilitators   - Speed - Convenience - Dependability of information - Satisfaction with information |
| Clinical practice guidelines (CPGs) as an information source | Treatment-related questions | Barriers   - Ineffective means of search - Lack of time - Frustration - Uncertain about continuity of care |
| Accessing online information using smartphones | Information on less common conditions |  |
| Patients as an information source |  |  |
| National electronic health record (NEHR) as an information source |  |  |
| UpToDate® as an information source |  |  |

**Subthemes and codes**

| **Information sources** | | |
| --- | --- | --- |
| **Codes** | **Definition (inclusion – keywords – and exclusion criteria)** | **Quotes** |
| **Colleagues as an information source** | | |
| Colleagues   - Pharmacist for medicine-related information - Senior | Clinicians reported seeking clinical information from various colleagues. | “I, actually called one of my colleague who actually did a dermatology diploma…To confirm my diagnosis” [Doctor, SIDRML09] |
| **CPGs as an information source** | | |
| In-house protocols | Clinicians reported using the organisation’s clinical practice guidelines and protocols for practice. | “…we have our own Intranet where we have the management protocols.” [Doctor, SIDRML08] |
| **Accessing online information using smartphones** | | |
| Online resources   - Patient reviews - Internet/Google/WhatsApp via smartphone   Guidelines via email | Clinicians mentioned the use of online resources and personal smartphones to seek clinical information. | “…internet, you have in your pocket straight…It’s in the handphone.” [Nurse, SINRML01] |
| **Patients as an information source** | | |
| Patients | Clinicians mentioned awaiting patients for information to inform practice. | “I have to decide or make a diagnose or come to a treatment plan based on the information they give.” [Doctor, SIDRML05] |
| **NEHR as an information source** | | |
| NEHR | Clinicians indicated accessing NEHR for confirmation of the patient’s medical condition, only when necessary, and for quick access to relevant information. | “The thing is that when I run the clinic, I don’t usually open the NHR automatically…Because you know, some people have some privacy concern and then without any… By right, without any…need for the information there, we shouldn’t be accessing…So, I make it a point to only open it when I think it’s necessary…so he told me he was here in KTP. Then I go in and see…Then I can get the information from the NHR then.” [Doctor, SIDRML06] |
| **UpToDate® as an information source** | | |
| UpToDate® | Doctors mentioned that UpToDate provided more comprehensive information on drugs than the organisation’s drug reference and that it was available as a mobile app. | “…my initial search was…Within the drug compendium…So, then I had to move on to UpToDate…So that I could have a more detailed information because the drug compendium did not give me much…clarity…Or did not allow me to make a decision straight-forward.” [Doctor, SIDRML05] |
| **Information needs** | | |
| **Codes** | **Definition (inclusion – keywords – and exclusion criteria)** | **Quotes** |
| **Immediate answers to clinical questions** | | |
| Patient management-related questions | Clinicians mentioned seeking information on the management of patients, such as social background, patient compliance, and more. | “…there were some on-going concerns whether there’s any…social circumstances that prevent adequate treatment or whether there was just treatment, pure medical treatment failure.” [Doctor, SIDRML18] |
| **Treatment-related questions** | | |
| Thought-provoking | Clinicians mentioned seeking clinical information to broaden one’s understanding. | “I think these are…more of, like…in depth knowledge base. Not the scraping the surface, then we tell the patient.” [Nurse, SINRML16] |
| **Information on less common conditions** | | |
| Had questions about patients with special considerations | Doctors indicated seeking clinical information for a special population. | “…today morning I had to see…a, pregnant lady who was on anti-hypertensives. So, I had to switch anti-hypertensives, which is not very oftenly done within the polyclinic clinic setting.” [Doctor, SIDRML05] |
| **Barriers and facilitators to information-seeking** | | |
| **Codes** | **Definition (inclusion – keywords – and exclusion criteria)** | **Quotes** |
| **Barriers to information access** | | |
| No access to the internet | Clinicians were not provided a connection to the internet by their organisation. | “Search engine no, I mean for now you want an immediate answer, you cannot go internet by the way” [Nurse, SINRML07] |
| High workload | Clinicians mentioned that the heavy burden at work made it hard for them to seek information. | “…it’s not easy to actually…explore more with a patient when you have little time and you have…the workload is a lot. Like, you know patients is waiting outside for you” [Nurse, SINRML16] |
| Slowness and unreliable internet connection | Clinicians indicated that unstable internet access limited their access to clinical information. | “Okay it can start, if it’s fast that means it’s…like internet connection very fast, it’s all good. If not, it’s frustration…Sometimes…disappointed if, let’s say…you try to get it and it just won’t work, like system down, that kind of thing.” [Nurse, SINRML07 |
| Ineffective means of search | Clinicians reported that the poor user interface of the medical website affected the speed of information retrieval. | “…whatever I can find from UpToDate, you know about the, about the latest management…Actually, even that took a while because…the website was not very… It’s not that compact. You have to go and read through a long list of things, right. So, about ten minutes.” [Doctor, SIDRML09] |
| Inability to access information due to IT | Clinicians displayed inadequacy in operating work computers to access clinical information. | “…oh this computer does not trust me.” [Doctor, SIDRML06] |
| No time to think | Doctors mentioned that they did not have the capacity to consider clinical questions. | “Because it’s a…Busy, busy clinic. No time to actually take a break to think.” [Doctor, SIDRML13] |
| Juggling multiple duties | Doctors reported having to manage several roles at work. Hence, unable to seek clinical information given the limited time. | “…juggle the timing because they’ve already put the patients on my list. So, the patient actually already outside my room…But I still have other duties to do. So, I can’t really do both the same time.” [Doctor, SIDRML11] |
| Feeling pressured by patients’ expectations and working as a public healthcare professional | Clinicians reported feeling stressed by the expectations from patients and working in a government-funded clinic. | “…so there’s a bit of…frustration as well in a sense…Because what happened is that…patients sometimes expect you to know everything, right?” [Doctor, SIDRML06] |
| Frustration and disappointment due to lack of access | Clinicians expressed dissatisfaction and anger when unable to have full access to clinical resources. | “…it also depends on whether you have the access. And then the restricted access, full article, partial article. And then if you don’t read the full article, you also don’t get the full picture. It gets a bit annoying as well.” [Nurse, SINRML02] |
| Struggling to recall information-seeking behaviour in the session | Clinicians had difficulty recalling what happened in their preceding clinical session. | “Hmm. Wah. This kind of thing…Suddenly think about it. Very hard. Let me think about it.” [Doctor, SIDRML06] |
| Lack of continuity of care for patients | Clinicians indicated limited nationwide healthcare work systems, such as limited sharing of patients’ health data across public and private health institutions affected patient care. | “…usually, I’ll go ahead and…ask them for permission to log onto NHR, because then there’ll be all the details there. But, because this patient is seeing another, private…GP…So there’s no way that I can check it.” [Doctor, SIDRML18] |
| Unfamiliarity with the concepts | Clinicians appeared to not understand the Evidence-based medicine concept. | “To the patient specifically or…regarding to the patient condition?” [Doctor, SIDRML18] |
| Workflow and IT issues affecting patient care   - Involving senior/trained colleagues to improve workflow | Clinicians indicated limited work systems, such as technical issues, and issues that needed to be escalated to the superiors were affecting patient care. | “…one was an IT issue…because I’m in a special room this morning that…With two results vetting as well as…jaundice patient…A baby with jaundice. So…I was supposed to be…Blocked out for a certain time so that I can do the vetting and the…baby jaundice…but I wasn’t, so, then I need to speak to boss and ask what happened...” [Doctor, SIDRML11] |
| **Facilitators to information access** | | |
| Speed of access | Clinicians reported taking less than five minutes when seeking clinical answers. | “…today it was very fast…Less than five minutes…It was sort of urgent.” [Doctor, SIDRML01] |
| Convenience | Clinicians tended to seek information when they perceived that the source was available and obtainable. | “I think the reason why going through colleague is my first…way of seeking information is because it’s the most convenient. It’s the laziest method. I mean, they’re just next door. You just ask them.” [Nurse, SINRML02] |
| Dependability of information | Clinicians mentioned the need to ensure that the clinical information was well-grounded and reliable. | “…we do call our own colleagues…Actually, we do have…doctors are quite senior…With more than 30 years of experience in the clinic. So, we do call the seniors.” [Doctor, SIDRML08] |
| Drive to confirm pre-existing knowledge | Clinicians reported being eager to know if their knowledge of the medical conditions were accurate. | “…the doctor is knowledgeable…But…we still have to…make sure that the things we’ve done are in line with the clinical” [Nurse, SINRML05] |
| Motivation | Clinicians expressed keenness to improve clinical knowledge, in learning and information seeking, as in the reasoning behind the clinical decisions made by colleagues for future practice. | “I would like to go back and read more…And I would like to make sure that…I am…my knowledge is more up-to-date so that I don’t have to go back to do a search the next time I encounter this problem.” [Doctor, SIDRML05] |
| Believed that information-seeking is a common behaviour | Clinicians thought that seeking clinical information for practice was normal practice among clinicians. | “It feels normal...Because searching for information is something that I always do.” [Nurse, SINRML16] |
| Easy access via the organisation’s network (intranet) | Doctors mentioned that UpToDate was accessible via work computers. | “And why did you choose UpToDate?...easy access…Yeah. We can access through intranet.” [Doctor, SIDRML30] |
| Organisation-provided subscription | Doctors reported the use of UpToDate medical resources as it was provided by their organisation. | “Where did you look for an answers to your questions is actually more, like…how would you look into?...UpToDate, because we subscribe to UpToDate.” [Doctor, SIDRML19] |
| Time | Nurses indicated answering clinical questions should time permit. | “I will on the train, then I will take out my phone, then I will Google” [Nurse, SINRML16] |
| Satisfaction when retrieving answers | Clinicians reported feeling satisfied while getting answers to their clinical questions. | “…fast, is good, you got certainty immediately and then you’ve got satisfaction.” [Nurse, SINRML07] |
