# Supplementary material for: Information‐seeking behaviour of primary care clinicians in Singapore at the point‐of‐care: A qualitative study
Source: Health Info Libr J. 2024 May 28;41(4):418–28. doi: 10.1111/hir.12535 (PMC11649596; doi:10.1111/hir.12535)
Supplement: Supplementary file 5 — Appendix S5. Supporting Information. [file HIR-41-418-s001.docx]

Multiple researchers, a reflexive journal, and documenting research assumptions and beliefs in the paper are all components of the reflexive research design. The analyst and perspective triangulation were demonstrated when at least two researchers carried out the thematic content analysis, and the types of questions were analysed using Ely and colleagues’ classification (1992).

| Individual reflexivity; diverse views that influence the study | I was the main researcher for this study. My experience has piqued my interest in how primary care clinicians use smartphones to access clinical data. To check up on clinical information during consultations, should clinicians utilise smartphones? I have to admit that, as a worried mother, I was unimpressed by the clinician's professionalism. I may have been biased towards clinicians utilising smartphones as an information tool when commencing this study. I was nonetheless reminded to avoid bias by the desire to conduct effective research, which includes conducting meaningful and legitimate interviews, presenting in-depth analysis data, and being a good researcher.  Minor instances were also documented in my field notes, which added to my reflexivity. For instance, I became anxious because I could not secure an alternative interview place when the interview room was last minute being used for another meeting or when the doctors and I overran our interview. Given that the interviews took place during the clinicians’ lunch break, and they were expected to return to the clinic for their patients promptly, I felt obliged to rush through the interview with the clinicians. Despite the delay, most clinicians were willing to take the time to complete the interviews. |
| --- | --- |
| Interpersonal reflexivity; Dynamics of power and interactions between study participants in the study | Clinicians often talked about their choice to take part in the study. Interestingly, when they realised the email containing the study's advertisement was from their supervisor, many felt pressured to participate. They looked worn out from their clinical duties, but they were ready to engage once the interview started.    I had never conducted both in-person and phone interviews before. I admitted that I was initially very nervous. I felt inadequate since I had to interview well-educated clinicians who would have dismissed me as a student. Would the response rate be satisfactory? Fortunately, another research team member oversaw some of the recruiting. As a result, I was relieved that my poor response rate was not due to my insufficiency. As a result, I was relieved that my poor response rate was not due to my insufficiency. My confidence grew as I felt prepared for the interviews. Reading the literature and attending seminars on coding and qualitative data analysis hosted by my institution's Family Medicine and Primary Care network helped me become acquainted with interview approaches. I also sought advice from people with substantial experience with interviewing and qualitative methods. Furthermore, contrary to my assumptions, I was thrilled to realise that clinicians were ready to assist me in recruiting other clinicians after they learned I was a PhD candidate. |
| Methodological; Methodology selection and its consequences | Semi-structured interviews were utilised to investigate participants' perspectives and attitudes on intricate and delicate subjects (Baumbusch, 2010). Utilising a uniform interview schedule was limiting due to the clinicians' varied occupations, educational backgrounds, and personal histories (Baumbusch, 2010). A semi-structured interview schedule acknowledges that not all participants use the same language and that not all words have the same meaning (Treece & Treece, 1986). This helps participants thoroughly comprehend a subject (Baumbusch, 2010).  No single analysis strategy is appropriate for all forms of interview data (Burnard, 1991). Interview data with more specific questions could also be analysed using a modified version of Burnard's technique (Burnard, 1991). Burnard's technique categorises, and codes interview transcripts in fourteen steps (Burnard, 1991). The COREQ is a widely accepted qualitative research reporting guide (Booth et al., 2014). It was designed to encourage careful and detailed reporting of interviews and focus groups. As a result, the COREQ checklist, which includes 32 criteria with precise descriptors, was employed in this study.  The study design I explored for this study had already been used in another study (Gorman & Helfand, 1995). This study design was described as the "after-visit interview, " meaning clinicians were interviewed after each clinic session or patient visit (Del Fiol, Workman, & Gorman, 2014). Despite the fact that after-visit interviews may have less influence on artificially stimulating queries, researchers may overlook queries clinicians fail to convey (Del Fiol et al., 2014). However, after-visit interviews may be completed more consistently, leading to more stable estimates of outcomes (i.e., the frequency of clinical questions clinicians had during the consultation) (Del Fiol et al., 2014). The self-report method is the least expensive and disruptive but is also the most susceptible to memory bias (Del Fiol et al., 2014). Nonetheless, given the logistical problems and high costs of direct observations and after-visit interviews, the self-report approach may be a viable option when the goal is to collect many clinical questions from various locations (Del Fiol et al., 2014). Since our research collaborators' privacy concerns prevented me from recording the clinical session, I chose to employ a semi-structured after-visit interview method to investigate the information-seeking behaviour of primary care clinicians in Singapore at the point-of-care. To ascertain whether primary care clinicians sought answers to clinical issues after clinical sessions, a second component of this study comprised follow-up interviews. Again, for individuals who agreed to be contacted again for additional study-related questions, quick follow-up semi-structured interviews with self-reported responses were done. These were incredibly helpful in figuring out whether primary care clinicians sought clarification on clinical questions after clinical encounters. |
| Excerpts from reflexive journal | |

References

Baumbusch, J. (2010). Semi-structured interviewing in practice-close research. *Journal for specialists in pediatric nursing, 15*(3), 255.

Booth, A., Hannes, K., Harden, A., Noyes, J., Harris, J., & Tong, A. (2014). COREQ (Consolidated Criteria for Reporting Qualitative Studies). In *Guidelines for Reporting Health Research: A User's Manual* (pp. 214-226).

Burnard, P. (1991). A method of analysing interview transcripts in qualitative research. *Nurse Educ Today, 11*(6), 461-466. doi:10.1016/0260-6917(91)90009-y

Del Fiol, G., Workman, T. E., & Gorman, P. N. (2014). Clinical questions raised by clinicians at the point of care: a systematic review. *JAMA Intern Med, 174*(5), 710-718. doi:10.1001/jamainternmed.2014.368

Gorman, P. N., & Helfand, M. (1995). Information Seeking in Primary Care: How Physicians Choose Which Clinical Questions to Pursue and Which to Leave Unanswered. *Medical Decision Making, 15*(2), 113-119. doi:10.1177/0272989X9501500203

Treece, E. M. W., & Treece, J. W. (1986). *Elements of research in nursing / Eleanor Walters Treece, James William Treece, Jr* (4th ed. ed.). St. Louis: Mosby.
