# Supplementary material for: Information‐seeking behaviour of primary care clinicians in Singapore at the point‐of‐care: A qualitative study
Source: Health Info Libr J. 2024 May 28;41(4):418–28. doi: 10.1111/hir.12535 (PMC11649596; doi:10.1111/hir.12535)
Supplement: Supplementary file 6 — Appendix S6. Supporting Information. [file HIR-41-418-s002.docx]

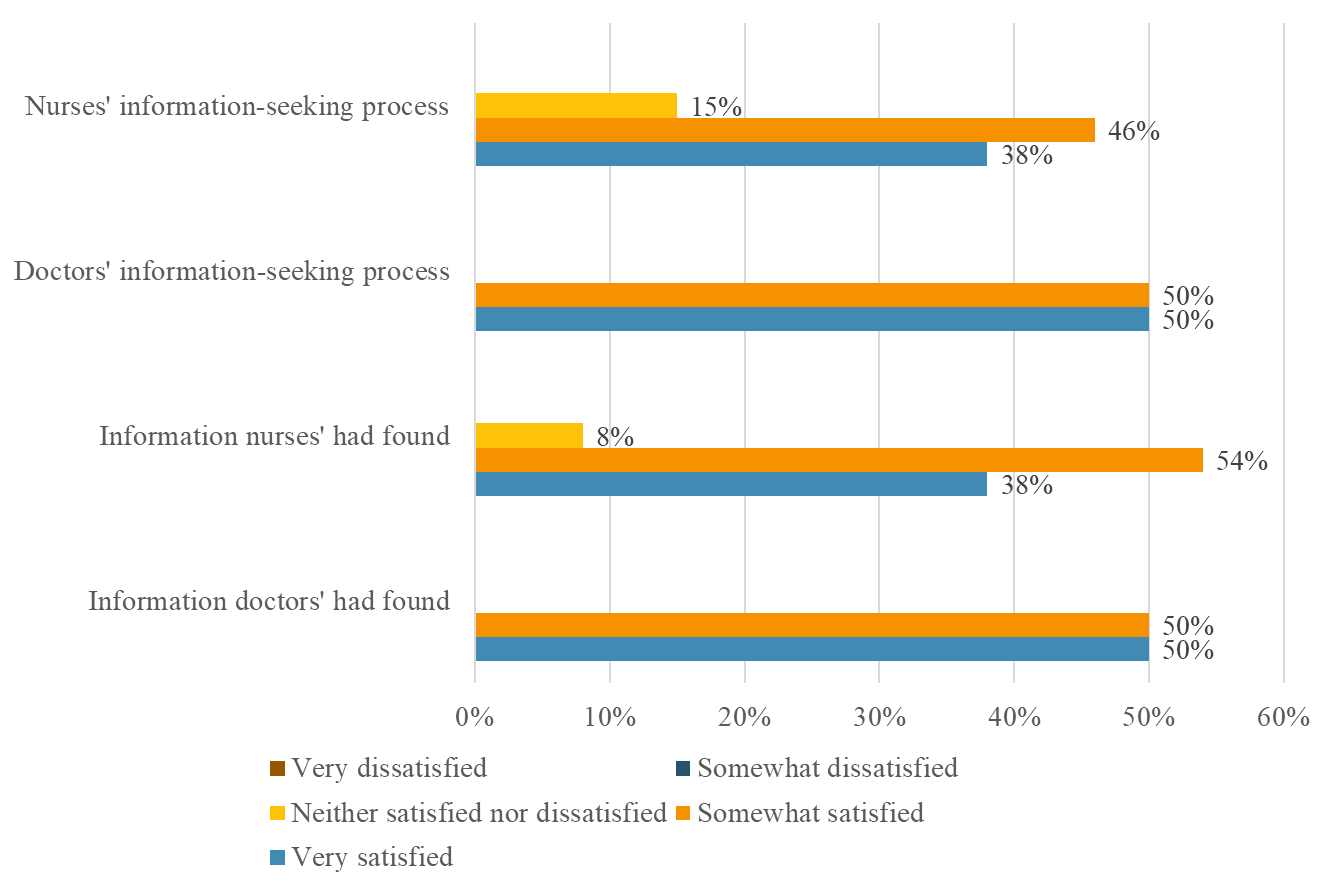


This figure shows the clinicians’ level of satisfaction in the information that they had found and their information-seeking process.
